# *Strobilanthes sunhangii* (Acanthaceae), a new species from Tibet, China

**DOI:** 10.3897/phytokeys.166.58831

**Published:** 2020-11-13

**Authors:** Jun-Tong Chen, Xian-Han Huang, Zhen-Yu Lv, Tian-Hui Kuang, Jian Luo, Yun-Fei Deng, Tao Deng

**Affiliations:** 1 Key Laboratory for Plant Diversity and Biogeography of East Asia, Kunming Institute of Botany, Chinese Academy of Sciences, Kunming 650201, Yunnan, China Kunming Institute of Botany, Chinese Academy of Sciences Kunming China; 2 University of Chinese Academy of Sciences, Beijing 100049, China University of Chinese Academy of Sciences Beijing China; 3 School of Life Sciences, Yunnan Normal University, Kunming 650092, Yunnan, China Yunnan Normal University Kunming China; 4 Institute of Plateau Ecology, Tibet Agricultural and Animal Husbandry University, Nyingchi 860000, Tibet, China Tibet Agricultural and Animal Husbandry University Nyingchi China; 5 Key Laboratory of Plant Resources Conservation and Sustainable Utilization, South China Botanical Garden, Chinese Academy of Sciences, Guangzhou 510650, Guangdong, China South China Botanical Garden, Chinese Academy of Sciences Guangzhou China

**Keywords:** Mêdog, morphological evidence, new species, *Strobilanthes sunhangii*

## Abstract

A new species of Acanthaceae, *Strobilanthes sunhangii*, is described from Mêdog County, Tibet, China. Morphologically, the new species is closely similar to *S. medogensis* and *S. divaricata*, but *S. sunhangii* differs in having glabrous stems, longer spikes, glabrous rachis, double curved corolla and glabrous calyx, different stamens and style.

## Introduction

*Strobilanthes*Blume (1826: 781) is one of the most diverse genera in the family Acanthaceae (Ruellieae: Strobilanthinae)and comprises approximately 450 species mainly distributed in tropical and subtropical regions of Asia with some species extending to Pacific Islands (Hu and Tsui 2002; Hu et al. 2011; Tripp et al. 2013; Chen et al. 2019; Deng 2019). Historically, there has long been disagreement about whether the genus might be adopted on a broad or narrow circumscription (von Esenbeck 1847; Anderson 1867; Clarke 1885; Terao 1983). Bremekamp (1944) divided Strobilanthinae into many small genera. However, molecular studies (Moylan et al. 2004) have shown that these segregate genera are nested within *Strobilanthes* and the broad concept of *Strobilanthes* has been generally accepted (Deng et al. 2006; Wood and Scotland 2009; Hu et al. 2011; Wood 2014; Deng 2019). In China, Hu et al. (2011) recorded 128 species mainly distributed in SE to SW China.

China has a vast territory with a wide range of complex and diverse topographies and soils and covering several climate types, which contribute to the wealth of Chinese botanical diversity (Sun et al. 2017; Chen et al. 2018a, b, 2020). Mêdog County, in which the highest peak is Mt. Namjagbarwa (7,782 m) in the north and the lowest point is Pasighat (154 m), has a rich biodiversity, including 1,805 species of seed plants (Sun and Zhou 1996, 2002; Yang and Zhou 2015; Chen et al. 2018b) and some new species have recently been reported from this region, such as *Cornus sunhangii* (Lv et al. 2019).

During a recent expedition in Mêdog, we discovered a species of *Strobilanthes* with very unusual morphological characters. After observations of wild living plants, herbarium specimens, laboratory studies and consultation of relevant literature (Blume 1826; Anderson 1867; Clarke 1885; Li 1985; Wood 1994, 2001, 2014; Carine and Scotland 2002; Hu and Tsui 2002; Deng et al. 2006; Hu et al. 2011; Adhikari 2018; Chen et al. 2019; Thomas et al. 2019), it is concluded that it does not match any other known species of *Strobilanthes* and represents an undescribed species. Therefore, it is described below as *Strobilanthes sunhangii* T. Deng, J.T. Chen & Y.F. Deng.

## Materials and methods

The specimens of *Strobilanthes sunhangii* were collected from Mêdog County in Tibet and studied at the herbarium of Kunming Institute of Botany (KUN). Morphological characters, recorded for the new species, were based on fresh plants and dried specimens. Pollen grains were collected from dry specimens and the operation method for pollen measurement followed Chen et al. (2019). Morphological comparisons of *S. sunhangii*, with the related taxa *S. medogensis* (H.W. Li) J.R.I. Wood & Y.F. Deng and *S. divaricata* (Nees) T. Anderson are provided in Table 1.

**Table 1.** Morphological comparisons of Strobilanthes sunhangii with related species.

Characters	*S. sunhangii*	*S. medogensis*	*S. divaricata*
**Stems**	Stems subterete, glabrous.	Stems slightly sulcate, bifariously puberulent.	Stems often zigzag above, angular, glabrous.
**Leaves**	Leaves slightly anisophyllous, leaf blade ovate to lance-ovate, smaller of pair ca. 2/3 size of larger one, 4.2–6.9 × 1.2–2.0 cm and larger ones 8.2–10.1 × 2.2–3.2 cm, base rounded to broadly cuneate, both surfaces glabrous and with numerous linear cystoliths.	Leaves anisophyllous, leaf blade ovate, smaller of pair ca. 2/3 size of larger one, (1.9–)4.0–6.0 × (0.5–)1.8–2.1 cm and larger ones 5.8–9.0 × 2.1–3.7 cm, base rounded to subcordate, both surfaces glabrous, adaxially with numerous cystoliths.	Leaves strongly anisophyllous, smaller leaves ca. 1/3 length of larger leaves, ovate, 1.5–6 × 0.5–2 cm, base rounded, larger ones lanceolate to elliptic, 8–18 × 2–5 cm, base cuneate or attenuate to a short petiole, both surfaces glabrous.
**Inflorescences**	Spikes slender, (7–)11–22 cm; rachis glabrous.	Spikes 3–6 cm; rachis bifariously pubescent.	Spikes 1–6 cm, rachis glabrous to sparsely pilose.
**Corolla colour**	Corolla outside and lobes pinkish-white, inside purplish-pink.	Corolla yellowish-white but dull purple on lobes.	Corolla purple.
**Corolla**	Corolla 2.8–3.3 cm long, tube basally cylindrical and ca. 3 mm wide by ca. 6 mm long, then bent to ca. 90° and gradually widened to 9–12 mm wide at middle by 16–18 mm long, then second bent to ca. 90° and tube upper cylindrical and 9–12 mm wide by 16–18 mm long.	Corolla ca. 2.5 cm long, straight, tube basally cylindrical and ca. 2 mm wide by ca. 1 cm long, then gradually widened to 1–1.5 cm at mouth.	Corolla 3–3.5 cm long, tube cylindrical at base for 1 cm, then gradually widened up to 0.8 cm at mouth, straight or slightly bent towards mouth.
**Corolla lobes**	Corolla lobes widely elliptic, ca. 8–9 × 7–8 mm, apices emarginate.	Corolla lobes broadly ovate, ca. 3 × 8 mm, apices rounded.	Corolla lobes 5–5.5 × 6 mm, apices obtuse or rounded.
**Calyx**	Calyx 7–8 mm, glabrous, 5-lobed to middle; lobes ovate, equal, margins narrowly scarious.	Calyx ca. 12 mm, gland-tipped pubescent, 5-lobed to base; lobes narrowly oblong-elliptic, subequal.	Calyx 8–15 mm, glabrous rarely pilose at tips, lobes linear-lanceolate, acuminate, subequal.
**Stamens**	Stamens included; filaments of shorter pair ca. 2 mm, longer pair ca. 4 mm; anther thecae oblong, ca. 4 mm.	Stamens exserted; filaments of shorter pair 7–8 mm, longer pair 9–10 mm; anther thecae ca. 1 mm, incurved.	Stamens included, filaments of shorter pair incurved, 1 mm, longer pair unequal, longer one 5–5.5 mm, shorter one 4–4.5 mm; anther thecae ca. 1 mm.
**Style**	Style ca. 1.8 cm, with sparse gland-tipped trichomes.	Style 2.7–2.8 cm, glabrous.	Style 2–3 cm, sparsely glandular trichomes at base.
**Distribution**	Tibet (Mêdog), China.	Tibet (Mêdog), China.	India (NE-India, Darjeeling,Sikkim); Bhutan; Nepal.

## Results

### Taxonomic treatment

#### Strobilanthes sunhangii

Taxon classificationPlantaeLamialesAcanthaceae

T. Deng, J.T. Chen & Y.F. Deng sp. nov.

3B42DDB7-0A21-5D02-8947-45755B5830D5

urn:lsid:ipni.org:names:77212877-1

Figs 1 , 2 , 3 , 4

##### Type.

China. Tibet: Mêdog County, Beibeng Town, 29.23319N, 95.17693E, elev. ca. 1,470 m, 5 Oct 2018, *H. Sun*, *T. Deng* & *Z.M. Li Sunhang19964* (***holotype***: KUN1345286!; ***isotypes***: IBSC!, KUN1345287!, KUN1345288!)

**Figure 1.**
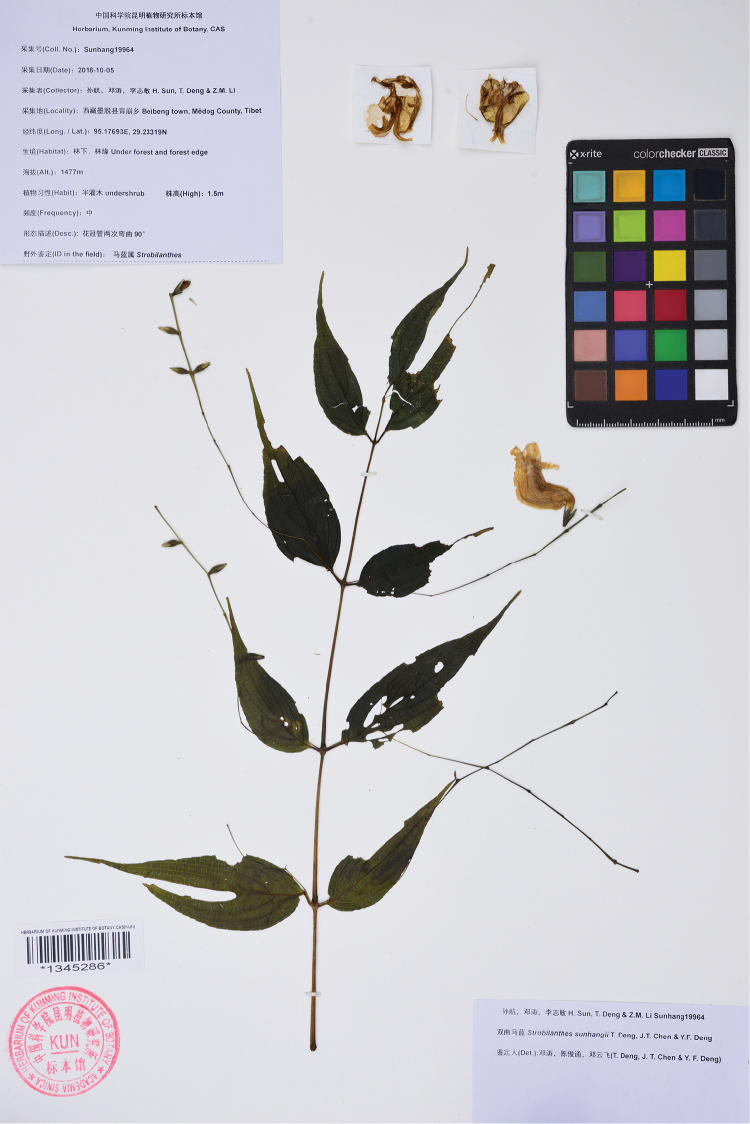
Photograph of the holotype of *Strobilanthes sunhangii* T. Deng, J.T. Chen & Y.F. Deng (KUN barcode 1345286!).

##### Diagnosis.

*Strobilanthes sunhangii* resembles *S. medogensis*, but differs by its stems subterete, glabrous (vs. slightly sulcate, bifariously puberulent); spikes (7–)11–22 cm long (vs. 3–6 cm long), rachis glabrous (vs. bifariously pubescent); corolla outside and lobes pinkish-white, inside purplish-pink (vs. corolla yellowish-white, but dull purple on lobes), the tube bent to ca. 90° twice (vs. straight), lobes apices emarginate (vs. rounded); calyx 7–8 mm long, glabrous, 5-lobed to middle (vs. ca. 12 mm long, gland-tipped pubescent, 5-lobed to base), lobes ovate, equal (vs. narrowly oblong-elliptic, subequal); stamens included (vs. exserted), filaments of shorter pair ca. 2 mm long (vs. 7–8 mm long), longer pair ca. 4 mm long (vs. 9–10 mm long), anther thecae ca. 4 mm (vs. ca.1 mm); style with sparse gland-tipped trichomes (vs. glabrous).

**Figure 2.**
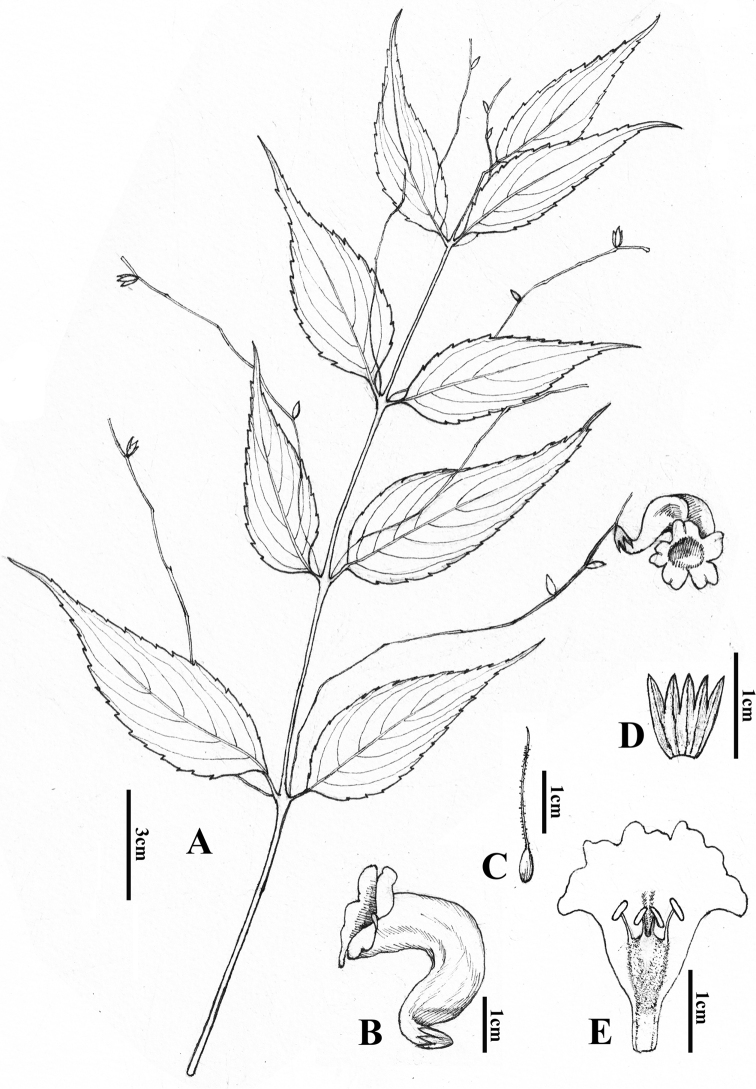
*Strobilanthes sunhangii* T. Deng, J.T. Chen & Y.F. Deng **A** plant showing flowering branch and leaves **B** flower (view from side) **C** pistil, ovary and style **D** calyx **E** opened corolla showing androecium (Drawn by Xiaoshuang Zhang based on the holotype of Sunhang19964).

##### Description.

**Figure 3.**
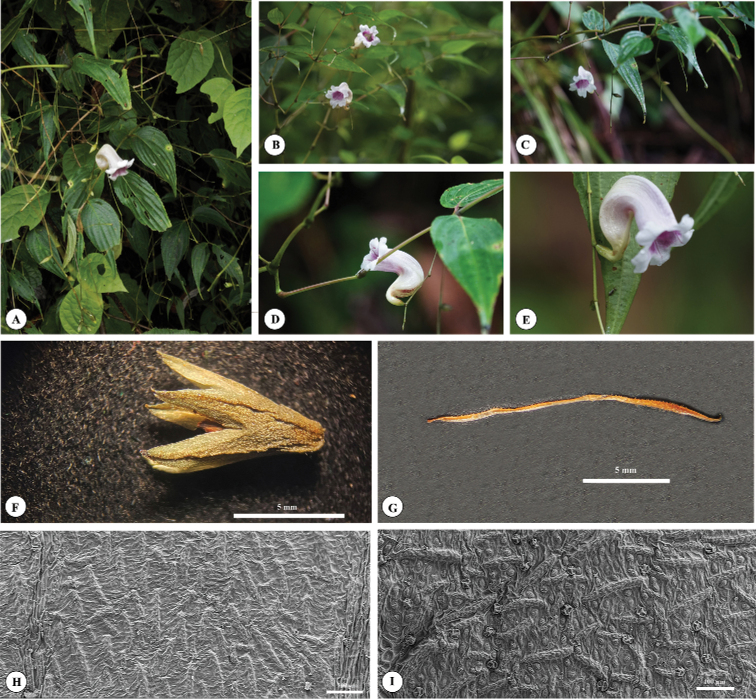
*Strobilanthes sunhangii* T. Deng, J.T. Chen & Y.F. Deng **A** habitat and flowering branch **B, C** flowering branch and flower (view from front) **D, E** flower (view from side) **F** calyx **G** style **H** leaf adaxial surfaces **I** leaf abaxial surfaces.

**Figure 4.**
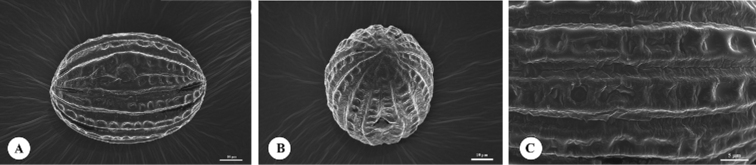
Pollen grains of *Strobilanthes sunhangii* T. Deng, J.T. Chen & Y.F. Deng **A** equatorial view **B** polar view **C** ornamentation.

Undershrubs 1–2 m high, gregarious. Stems subterete, glabrous, slightly swollen at node. Leaves slightly anisophyllous, smaller one ca. 2/3 of the larger one in size; petiole 3–7 mm long or petiole of smaller leaf in each pair shorter, sulcate, glabrous; blades ovate to lanceolate-ovate, smaller ones 4.2–6.9 × 1.2–2.0 cm, larger ones 8.2–10.1 × 2.2–3.2 cm, both surfaces glabrous, densely covered with numerous linear cystoliths, secondary veins 3–5 on each side of midvein and prominent on both surfaces, base rounded to broadly cuneate, slightly oblique, margin serrulate, apex acuminate and narrowly caudate. Inflorescences of axillary spikes, simple or rarely 2-furcate, slender, (7–) 11–22 cm long; rachis glabrous; bracts and bracteoles not seen, caducous. Flowers (0.6–) 1.0–2.5 (–3.0) cm apart on rachis. Calyx 7–8 mm long, glabrous, 5-lobed to middle; lobes ovate, equal, margins narrowly scarious, 1-nerved abaxially, conspicuous after drying, apices acuminate. Corolla 2.8–3.3 cm long, campanulate, outside and lobes pinkish-white, inside purplish-pink, outside glabrous, inside glabrous except for bifarious hirsute retaining the style (Fig. 3E); tube basally cylindrical, ca. 3 mm wide and ca. 6 mm long, then bent to ca. 90° and gradually widened to 9–12 mm wide at middle and 1.6–1.8 cm long, then secondly bent to ca. 90° and tube upper cylindrical 9–12 mm wide and 1.6–1.8 cm long; lobes broadly elliptic, 8–9 × 7–8 mm, apices emarginate (Fig. 3A, B). Stamens 4, didynamous, included; the united part of filaments glabrous at base, with dense villous on middle and margin, with slightly sparse villous on upper (Fig. 3E), shorter pair ca. 2 mm long, longer pair ca. 4 mm long; anther thecae oblong, ca. 4 mm long. Pollen grains prolate in outline, 74.1 (72.2–76.1) × 49.7 (48.1–50.4) μm, P/E = 1.49, tricolporate, ribbed pseudocolpi 12, exine reticulate (Fig. 4). Ovary ca. 5 mm long, glabrous; style ca. 1.8 cm long, with sparse gland-tipped trichomes. Capsule not seen.

##### Phenology.

Flowering from June–October.

##### Etymology.

*Strobilanthes sunhangii* is named after Prof. Hang Sun (1963–) for his outstanding contributions to the flora of Himalayan regions and collecting this new species for the first time. Verucular name: The Chinese name is given as “双曲马蓝” (shuāng qū mǎ lán), referring to the double-curved corolla of the new species.

##### Distribution and habitat.

The new species is currently known only from Mêdog County, Tibet, China. It grows in the moist evergreen forest at an elevation of 1,200–1,800 m. The associations include *Exbucklandia populnea* (R. Br.) R. W. Brown (Hamamelidaceae), *Viburnum* sp. (Viburnaceae), *Rubus* sp. (Rosaceae), *Campanumoea inflata* (Hook. f. & Thomson) C.B. Clarke (Campanulaceae), *Tripterospermum volubile* (D. Don) H. Hara (Gentianaceae) and *Impatiens* sp. (Balsaminaceae), amongst others.

##### Conservation status.

The new species was only discovered in Beibeng Town, Mêdog County. About 1,000 individuals were observed and the extent of occurrence was ca. 5 km^2^. Further explorations to obtain the precise population status are needed to assess its conservation status. Based on available data, the new species is assigned to the category ‘Data Deficient’ (DD) of the International Union for Conservation of Nature (IUCN 2019).

##### Specimens of *S. medogensis* examined.

– China. **Tibet**: Mêdog County, Gedang Town, elev. ca. 2,400 m, 27 Aug1974, *QTP Expedition 74-4911* (holotype: KUN barcode 1219209!); Mêdog County, Gedang Town, 2,450 m elev., 11 Sept 1982, *B.S. Li & S.Z.Cheng 814* (PE); Mêdog County, Gedang Town, 1,940 m elev., 13 Oct 1982, *S.Z. Cheng & B.S. Li 1457* (PE); Mêdog County, 1,100 m elev., 1 Dec 1982, *S.Z. Cheng & B.S. Li 3238* (PE); Nyingchi County, Pailong Town, 2,175 m elev., 8 Sept 2006, *Tiber-MacArthur 261* (A); Lung County, Chayne chu, 2,750 m elev., 10 Jul 1936, *Ludlow & Sherriff 2336* (BM, E).

##### Specimens of *S. divaricata* examined.

– India. **Meghalaya**: Khasia, 1,524 m elev., 5 Oct 1886, *C. B. Clarke 45219* (BM, CAL, LE); Khasia, 1,219 m elev., 12 Nov 1871, *C. B. Clarke 15479* (CAL, K, LE); Khasia, Oct 1850, Simons 482 (CAL). **Sikkim**: Khuping, 1,676 m elev., 26 Sept1884, *C. B. Clarke 35904* (LE); Khuping, 1,676 m elev., 26 Sept 1884, *C. B. Clarke 35920* (LE). – Nepal. Koshi Zone, Sankhuwasabha District, 2,020 m elev., 16 Aug 1998, *S. Noshiroet al. 9840046* (A); Dhawalagiri Zone, Myagdi District, 1,820–2,360 m elev., 10 Sept 1996, *M. Mikage et al. 9681329* (A).

## Discussion

Morphologically, the new species is closely related to *Strobilanthes medogensis* in having similar leaves and slender spikes, but differs in having glabrous stems, longer spikes, glabrous rachis, double curved corolla and glabrous calyx, different stamens and style (Li 1985; Wood 1994; Hu and Tsui 2002; Deng et al. 2006; Hu et al. 2011). In addition, the new species is also related to *S. divaricata* in the glabrous stems and calyx, slender spikes, but differs in having longer spikes, double curved corolla, different stamens and style (Wood 2001; Adhikari 2018). The new species is quite different from all other known species in its unique corolla shape. The corolla of *S. sunhangii* are bent twice, at the base and middle of throat, respectively, while that of other species is straight or curved at the middle of the throat. The detailed morphological comparisons amongst *S. sunhangii*, *S. medogensis* and *S. divaricata* are shown in Table 1.

Till now, no infrageneric classification has been established. Bremekamp (1944) divided the subtribe Strobilanthinae into 54 genera and 27 informal groups. However, his classification is not supported by the studies of the only molecular phylogeny of *Strobilanthes* (Moylan et al. 2004) and morphological analysis (Carine and Scotland 2002). In this study, *S. divaricata* and *S. medogensis*, two species closest to the new species, were not included. Bremekamp placed *S. divaricata* into his new genus *Diflugossa* Bremek., which is characterised by the single flowers on each node on rachis, arranged in an open panicle, bracts small, deciduous and corolla straight. However, *S. divaricata* is quite different from other species of *Diflugossa*, which was informally placed in Panicle groups by Wood and Scotland (2003), in its paired flower on the rachis, arranged in spikes. *S. medogensis* was placed in *Goldfussia* Nees, which is characterised by the flowers arranged in congested head-like spikes and then compounded into small panicles, stamens 2-paired, the shorter pair unequal, nodding, anthers spherical (Li 1985). Obviously, these three species form a species complex and further studies on the placement in the genus are necessary, depending on the establishment the infrageneric classification.

Pollen morphology has been widely used in species delimitation of *Strobilanthes* (Chen et al. 2019), so we observed and measured the pollen grains of this new species. Pollens grains of the new species are prolate, 74.1 (72.2–76.1) × 49.7 (48.1–50.4) μm, P/E = 1.49, 3-colporate, 12-pseudocolpate, with reticulate exine. The detailed pollen grains morphology is provided in Fig. 4. The pollen grains of *S. medogensis* (as *S. campaniformis*) were observed by Wood (1994) and those of *S. divaricata* have not yet been observed. Both *S. sunhangii* and *S. medogensis* belong to type 3 recognised by Hu et al. (2011). Therefore, the close relationship between *S. sunhangii* and *S. medogensis* is also supported by the pollen morphology.

This species was observed by the authors during the Mêdog expedition in 2018. To date, we only collected flowering specimens and whether this new species has a plietesial life history requires further explorations.

## Supplementary Material

XML Treatment for Strobilanthes sunhangii
